# Clinicopathological spectrum of solitary Plasmacytoma: a single center experience from coastal India

**DOI:** 10.1186/s12885-019-5976-7

**Published:** 2019-08-14

**Authors:** Sridevi Hanaganahalli Basavaiah, Flora D. Lobo, Cheryl Sarah Philipose, Pooja K. Suresh, Saraswathy Sreeram, Hema Kini, Kausalya K. Sahu, Krishna Prasad

**Affiliations:** 1 Department of Pathology, Kasturba Medical College Mangalore, Manipal Academy of Higher Education, Manipal, Karnataka 575001 India; 2 Medical Oncologist, Mangalore Institute of Oncology, Mangalore, Karnataka India

**Keywords:** Plasma cell disorders, Extramedullary, Solitary

## Abstract

**Background:**

Plasma cell disorders are a rare group of hematological malignancies that accounts for 10% of all hematological neoplasms. Solitary plasmacytomas are rarer entities accounting for less than 5% of all the plasma cell dyscrasias. They encompass three subtypes - Solitary Plasmacytoma of Bone (SPB) and Solitary Extramedullary Plasmacytoma (SEP) and multiple solitary plasmacytomas (MSP). In this study, we discuss the clinical, histopathological and immunohistochemical characteristics of solitary plasmacytomas.

**Methods:**

A 13 year retrospective analysis of solitary plasmacytomas was performed from a single tertiary care center. Bone marrow evaluation was done concurrently at the time of diagnosis to rule out the presence of multiple myeloma.

**Results:**

A total of 29 cases fulfilled the diagnostic criteria for SP during the study period. SPB accounted for 55.2%, SEP for 44.4% and MSP for 3.4% of the cases. The most common sites involved were the paranasal sinuses and vertebrae. Other infrequent sites included lymph node, tonsil and lungs. The mean age of presentation of SPB was a decade later than SEP. A male preponderance was observed in both subtypes.

**Conclusion:**

Solitary plasmacytoma is a rare entity, the diagnosis of which requires a systematic approach. There is limited data available in the literature on the clinico-pathological characteristics of SP from India.

## Background

Plasma cell dyscrasias are neoplastic proliferations of monoclonal plasma cells that encompass a wide range of entities. At one end of the spectrum, lies Monoclonal Gammapathy of Undetermined Significance (MGUS) which is a silent disorder, while at the other end lies the more aggressive entity, multiple myeloma (MM) [1]. An estimated incidence of myeloma in India is 6800 new cases every year with an age standardized rate of 0.7/100,000 population and a 5 year prevalence is 1.4/100,000 population amounting to 11,600 cases [2]. The estimated mortality rare from MM in India accounts for around 5900 deaths every year. The median overall survival (OS) when treated with a combination of alkylating agents (Thalidomide, Dexamethasone and Bortezomib) varies between International Staging System (ISS) groups. In India, the median OS is 55 months, 48 months and 27 months for ISS I, II and III groups as reported by Jacob et al. in 2017 [3]. Myeloma occurs most commonly in the sixth decade of life in Indian subcontinent, as reported by Kaur et al., and the reported male:female ratio is 1.4:1 with a clear male preponderance [4, 5].

Solitary plasmacytomas (SP) are rare neoplasms, that often occur with the absence of systemic manifestations and bone marrow involvement, but do have a propensity to eventually progress to MM [6]. SPs account for 1–5% of all plasma cell neoplasms [6, 7]. SP may be either solitary plasmacytoma of the bone (SPB), solitary extramedullary (extra-ossesous) plasmacytoma (SEP) or multiple solitary plasmacytomas (MSP) [6, 8]. In SPB, any bone may be involved, but bones that actively produce hematopoietic elements such as pelvis, skull, ribs, spine, femur, clavicle and scapula are more commonly involved [6, 8]. The plasmacytomas are termed as MSPs when there are multiple sites of involvement in soft tissue, bone or both without any evidence of increased plasma cells on a random iliac crest bone marrow biopsy, associated with absence of hypercalcemia, renal failure or serum monoclonal light chains [9]. SEP tend to occur at sites other than bone such as the gastrointestinal tract, the respiratory tract, bladder, thyroid, kidney, pancreas, uterus or the central nervous system [8]. Although, they are histologically similar to MM, albeit are associated with a better prognosis [6]. In particular, soft tissue plasmacytomas are at a lower risk of transformation to MM and are treated with chemoradiotherapy [1, 6].

Besides a 10 year study from North India conducted at AIIMS, New Delhi, there are no similar large scale studies on SP from the Indian Subcontinent [10]. To the best of our knowledge, although many case reports have been documented in literature, the present study relating to the data on SP, is probably the first of its kind from Southern India. We aim to study the clinico-pathological characteristics and management of Solitary Plasmacytoma, an experience from a single institute of South India.

## Methods

The study was approved by institutional ethics committee. Informed consent from the participants was waived by IRB due to the retrospective nature of study that involved the slides of lesions without direct interrogation with the patients. In a retrospective study performed in the Department of Pathology of a tertiary care center, over a duration of 13 years from January 2006 to December 2018, all cases of SP diagnosed by histopathology were collected. The initial work-up of the cases included clinical history and physical examination; hematological investigations such as complete blood count, peripheral smear and bone marrow examination; biochemical investigations such as blood urea nitrogen, serum calcium, serum protein electrophoresis, estimation of free light chains, as well as a radiological skeletal assessment. The light chain assessment was performed using immunoturbidometry method. All the cases that fulfilled the diagnostic criteria recommended by Soutar R et al. were included in the present study [11]. The authors recommended the following criteria for the diagnosis of either SPB or SEP:

1. Single lytic bone lesion (in case of SPB) or extramedullary mass lesion (in case of SEP), that is histologically composed of clonal plasma cells
2. Normal bone marrow aspirate and biopsy evaluation, without any clonal plasma cell population
3. Absence of bone involvement, excluding solitary lesion, on skeletal survey or MRI study of spine and pelvis
4. Absence of end organ damage, such as CRAB (Hypercalcemia, Renal impairment, Anemia and Multiple osteolytic lesions) attributable to plasma cell dyscrasia

The criteria to diagnose MSP includes monoclonal plasma cell proliferation involving one or more lytic lesions of bone often spreading to the adjacent soft tissue, without evidence of bone marrow involvement on a random iliac crest bone marrow biopsy, and absence of evidence of end organ damage (hypercalcemia, renal failure, anemia, monoclonal proteins in the urine/serum or monoclonal light chains) [9].

At initial presentation, a bone marrow aspiration and trephine biopsy were carried out to rule out associated plasma cell myeloma. The cases in which a bone marrow study was not performed or report was unavailable, were excluded from the present study. The demographic data, history, radiological reports, laboratory results and other relevant details of each case were retrieved from the patient’s case files. The histopathological and immunohistochemistry sections of all the cases were retrieved and reviewed.

## Results

In a total of 130,496 histopathological specimens reported during the study period, plasmacytomas accounted for 57 cases. Three cases, which were received as slides for opinion, lacked complete clinical details and thus were excluded. Due to involvement of bone marrow by myeloma, or lack of either bone marrow status at the time of diagnosis or tracing the clinical data in the medical archives, 25 cases were further excluded. A total of 29 cases fulfilled the inclusion criteria and formed the core of the study. The present study was conducted in a hospital which is a tertiary referral center and receives cases from across most parts of South-West Coastal India that covers varied population and hence the true incidence of SP cannot be calculated. However, the catchment population of South-West Coastal India may approximately vary from 2 to 3 million. The proportion of SP based on the total number of histopathological specimens received was found to be 0.02%. These cases were further categorized into SPB (16 cases), SEP (12 cases) and a case of MSP of bone. The demographic data is depicted in Table 1. The age of the patients ranged from 27 to 83 years (mean- 56.7 years). A male predominance was noted with M:F ratio of 1.9:1.

**Table 1:** Age and gender distributions of solitary plasmacytomas

Plasmacytomas	Number of cases	Mean Age (range) in years	M:F ratio
Solitary Extramedullary Plasmacytoma	12	51.1 (27–72)	2:1
1. Paranasal sinuses	6	50.2	2:1
Maxillary sinus	3	62.7	All males
Frontal sinus	1	38	F
Sphenoid sinus	2	37.5	1:1
2. Lung	1	45	F
3. Lymph Node	2	57	All males
4. Oral Cavity	3	51	2:1
Tonsil	1	48	M
Gingiva	1	60	F
Buccal vestibule	1	45	M
Solitary Plasmacytoma of Bone	16	60.8 (40–83)	1.7:1
1. Vertebral body	5	64.4	1.5:1
Cervical	1	65	F
Thoracic	2	75.5	All males
Lumbar	2	53	1:1
2. Scalp	1	40	M
3. Clavicle	1	61	M
4. Sternum	1	62	M
5. Rib	1	70	M
6. Pubic bone	1	43	M
7. Femur-Greater trochanter	1	77	M
8. Hard palate	1	58	F
9. Right zygomatic arch	1	60	F
10. Anterior Chest wall	3	59.7	0.5:1
Multiple Solitary Plasmacytoma of bone	1	60	M

**Fig. 1:**
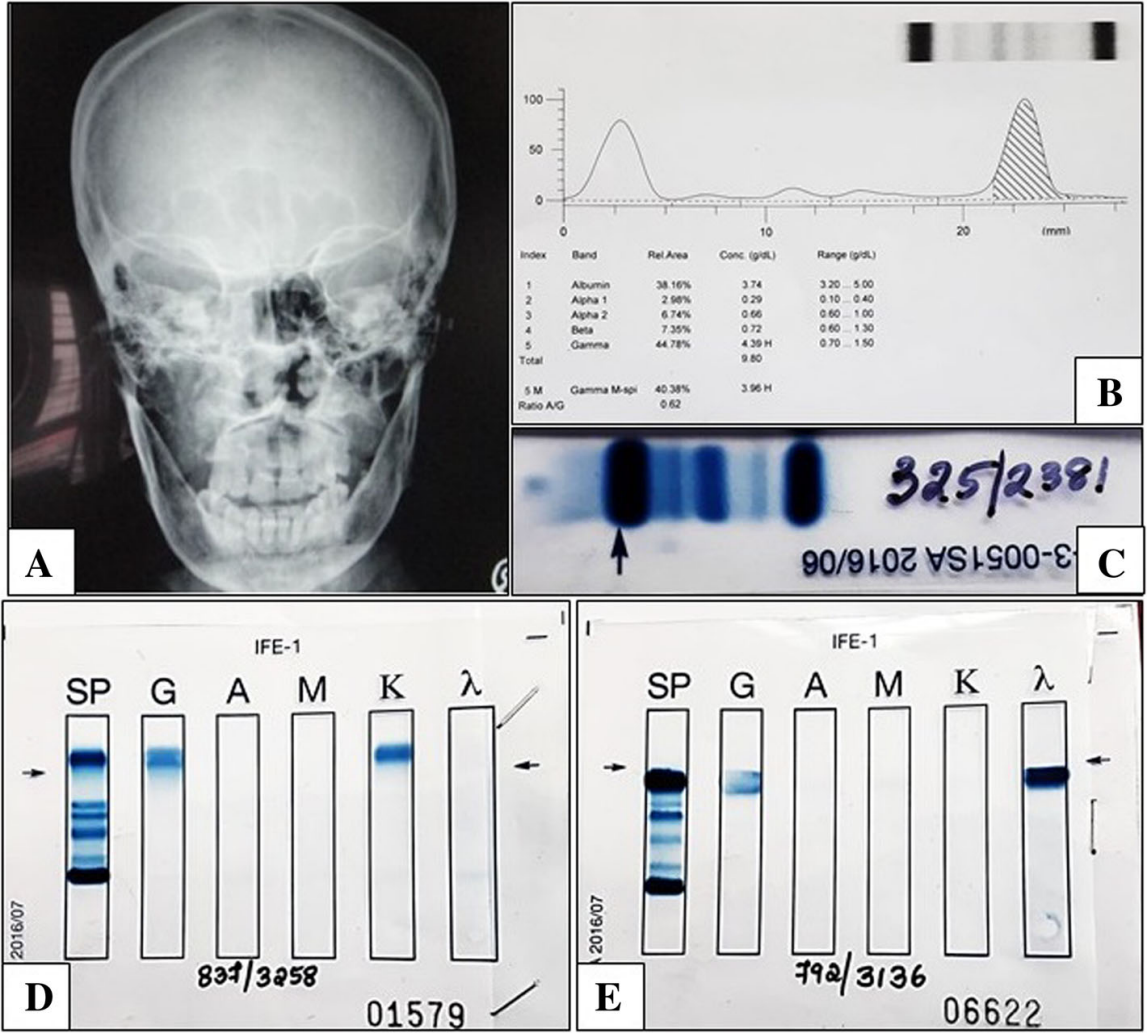
**a** Radiograph of plasmacytoma of right paranasal sinus presenting as a destructive osteolytic lesion (PA view) **b** & **c**: Serum protein electrophoresis with a significant M band suggecting paraproteinemia. **d** A case of SP with monoclonal kappa detected on IFE. **e** A case of SP with monoclonal lambda detected on IFE

Of the various sites, paranasal sinus was most frequently involved. It constituted for 22.2% cases of solitary plasmacytomas overall and accounted for 50% cases of SEPs (Fig. 1a). Among the paranasal sinuses, maxillary sinus was affected frequently (10.3%). Among the SPBs, the vertebra was affected more often (17.2%). The most common presentation of patients with SPB was pathological fracture (43.8%), whereas patients with SEP cases presented with soft tissue masses (91.7%). In the aero-digestive tract, these cases usually presented as polyps (50%), while in solid organs such as the lungs, space occupying lesion was more frequently encountered. Sino-nasal SEPs frequently presented with obstructive symptoms (100%), episodes of epistaxis (33.3%) or rhinorrhea (16.7%). A patient who presented with SEP in the sphenoid sinus has signs of right eye ptosis and 3rd cranial nerve palsy.

The clinical differential diagnoses considered were primary malignancy of the affected region, metastasis from unknown primary, lymphoma, tuberculosis, round cell tumor, fibrosarcoma, giant cell tumor, osteosarcoma or an inflammatory masses. It was often observed plasmacytomas of paranasal sinus were often mistaken for papillomas, particularly when cases presented as polypoidal masses.

Monoclonal (M) protein was present in 31.3% cases of SPB and 41.7% cases of SEP (Fig. 1b-e). On imaging of the skeletal system, the SPB cases were localized, well demarcated and lytic in nature. There was absence of multiple lytic lesions in the bone except in the case of MSP which had involvement of L3 vertebra, right 10th rib, right scapula, bilateral shoulder and posterior skull. The case of MSP had a previous history of posterior stabilization with decompression of L1 to L5 vertebra and was further evaluated for MM. There was no evidence of end organ damage with calcium-8.7 mg/dL, serum albumin-3.2 g/dL, total protein-5.6 g/dL, A:G ratio-1.85, urine Bence Jones protein- negative, ESR-8 mm/hr., hemoglobin-12.8 g/dL, LDH-179 U/L, serum creatinine-0.6 mg/dL, serum urea-18 mg/dL and free light chain assay and ratio were within normal limits. The iliac crest bone marrow aspiration and biopsy showed 3% plasma cells with no evidence of myeloma. The lesional biopsy from L3 vertebra showed monoclonal plasma cell proliferation with neoplastic plasma cells that were kappa restricted and positive for CD138 and negative for CD45. Possibility of non-Hodgkin lymphoma with plasma cell differentiation was ruled out with further immunohistochemical markers.

**Fig. 2:**
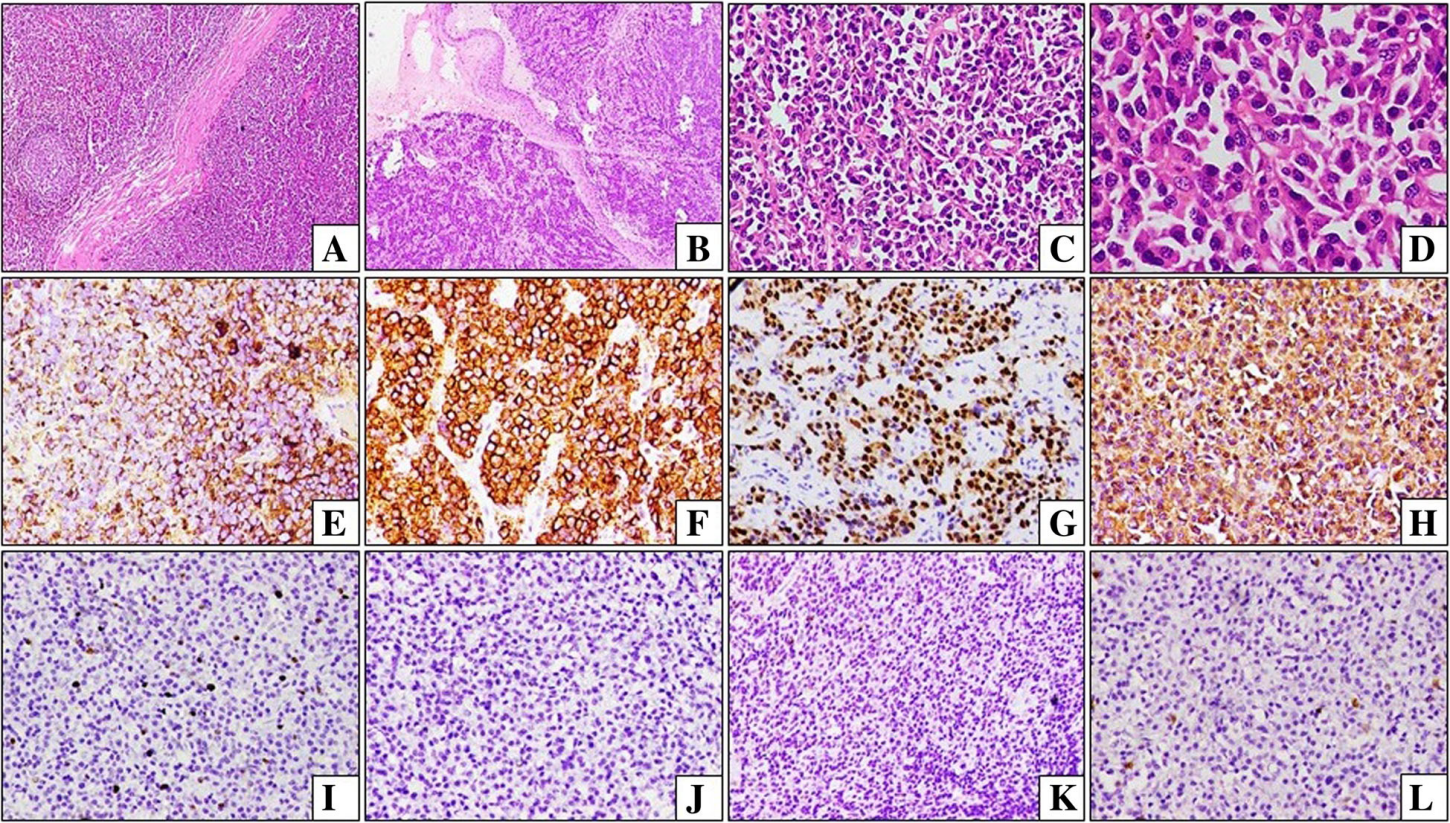
**a** Solitary Extramedullary plasmacytoma of lymph node: Lymph node architecture is partially effaced with few residual follicles and is infiltrated by neoplastic cells. **b** Solitary Extramedullary plasmacytoma of tonsil: Neoplastic infiltration noted on either side of the tonsillar cleft that is lined by intact squamous epithelium. **c** Neoplastic plasmacytoid cells in diffuse sheets. **d** Neoplastic cells with eccentrically placed round to oval nuclei and vesicular chromatin. Perinuclear halo are noted in some. **e** A case of SEP with monoclonal lambda positivity. **f** A case of SEP with monoclonal kappa positivity. **g** Neoplastic cells with MUM1 positivity. **h** Neoplastic cells with diffuse CD138 positivity. **i** Low proliferative index in neoplastic cells with Ki-67 immunomarker. **j** Negative LCA expression. **k** Negative CD20 expression. **l** Negative CD3 expression

Hematological work-up including bone marrow examination of all the cases ruled out multiple myeloma at the time of presentation. The average plasma cell percentage in the bone marrow was 8.2%. Histopathological study of the lesional biopsy showed a monotonous population of neoplastic plasma cells with eccentric nuclei, perinuclear hof and abundant basophilic cytoplasm (Fig. 2a-d). On immunohistochemistry (IHC), the tumor cells were CD45 and CD20 negative, and expressed CD138 and Epithelial membrane antigen (EMA). The neoplastic cells were monoclonal with surface expression of either kappa or lambda (Fig. 2e-l). Aberrant CD45 positivity was noted in a 65 year old patient with vertebral body plasmacytoma.

## Discussion

Plasmacytoma, first described by Schridde in 1905, is a rare entity and is defined as a localized mass of neoplastic monoclonal plasma cells [12]. They are not associated with systemic manifestations of MM and SPB is commoner than SEP. The commonly involved bones include the vertebra and skull. SEP is encountered more frequently in head and neck, in which nasal cavity and nasopharynx are by far the most common sites [6]. According to the literature, SPB is commonly seen in marrow containing bones and SEP is commoner in sites having a rich lymphatic drainage such as upper respiratory tract [6, 8, 13]. In the present study, vertebra was the commonest site for SPB and paranasal sinuses was the commonest site of SEP. In a similar study by Purkayastha et al. [8], the incidence of SEP in the head and neck was reported to be less than 1%. The nasal cavity, nasopharynx, paranasal sinuses, larynx and oropharynx were other commonly encountered sites that were reported by the authors [8].

In the present study, we encountered unusual sites of plasmacytomas such as lymph nodes, lungs and tonsils. Primary SEP of lymph node is extremely unusual and only a few case reports are documented in the literature [14, 15]. Most patients of lymph node SEP have indolent clinical course but slow progression to MM has nevertheless been documented [15]. There are only 11 cases of primary pulmonary plasmacytoma reported up to 2016 [16]. These cases generally benefit from surgical excision with or without radiotherapy. However, any case with diffuse parenchymal infiltration or those presenting with multiple nodules may require adjuvant chemotherapy. Tonsils are yet another rare site of SEP with only 5 cases reported up to 2015. Tonsillar SEPs can be treated with surgical excision and radiotherapy but there is no distinctive advantage of adjuvant chemotherapy in these patients [17].

Since SPB and SEP are rare tumors, understanding of the pathobiology of these elusive tumors are based on retrospective analysis of series of patients available in the literature, by a subgroup of Guidelines Working Group of the UK Myeloma Forum [11]. The researchers came to a consensus and listed the various levels of evidence and grades of recommendations for diagnosis and management of SPs that has been subsequently published by Soutar et al. [11].

The age bracket of SP ranges from 55 to 60 years and the incidence rises exponentially with advancing age [4]. However, as compared to MM, the occurrence of SPs is less common in the older age group [6, 18]. In the present study, the mean age at presentation for SPB was comparatively older (60.8 years) than that for SEP (51.1 years). We observed similar trends of an increase in the number of cases with advancing age. Only one case was detected in the 3rd decade of life and 13 cases (44.8%) observed in the 7th decade. There were only three cases in the 8th decade and one case in the 9th decade. Previous studies have shown a male preponderance and a finding that concur with present study [6, 18]. However, the M:F ratio was higher in SEP (2:1) as compared to SPB (1.7:1).

Skeletal survey by either CT, PET/CT or MRI is necessary not only for evaluation of the lesion but also to rule out additional lesions [11]. PET scan has been used as a diagnostic tool to stage MM and SPs. Similar to MRI, PET will aid in identifying occult disease in patients with SPB. Zhang et al. studied the utility of PET/CT in managing cases of suspected SEP. The authors concluded that PET/CT was helpful in identifying the additional lesions including involvement of lymph nodes, which has direct impact on the choice of treatment modality [19]. The use of CT, MRI or PET/CT in evaluation of plasma cell dyscrasia has increased the chances of detecting multiple soft tissue or additional bone lesions, thus accurately diagnosing cases of MSP when biochemical and laboratory investigations are within normal limits [9, 19, 20]. In the present study, different radiological modalities were used for workup based on circumstances.

Majority of the cases of MSP reported has only one bone lesion. However, there are case reports where multiple bone involvement are noted. Dattolo et al. [9] has described a case of MSP that involved two vertebrae (D2 and D3) and three ribs, while Kulbacki et al. [21] reported a case that involved C1 vertebrae, left first rib and hip bone. Collier et al. presented a case of MSP in an adult man who presented with increased intracranial pressure who on evaluation had lytic lesions of the skull, sacrum and left clavicle [22]. Ooi et al. has described 6 cases of MSP, 4 cases of multiple SEP and 2 cases with involvement of both bone and extramedullary sites [20]. It is important to distinguish cases of MSP from MM as the treatment and prognosis are different. Similar to all cases of MSP described in the above case reports and series, the case of MSP in our series did not have any evidence of paraproteinemia, urinary Bence Jones protein, renal involvement or bone marrow involvement ruling out the possibility of MM. MSPs are rare and have heterogeneous presentations, and also lack standard guidelines for its treatment. Experience of treating MSP are found in single or multicentric retrospective case series where chemotherapy, radiotherapy and surgical excision have been tried with variable response [9]. In our series, patient with MSP was started on chemotherapy but was lost to follow up after the first cycle.

Presence of M protein in SP, at the time of presentation, has been described in previous studies [23]. Compared to protein electrophoresis, immunoelectrophoresis increases the chances of detection of M protein. We found M protein in both SPB and SEP cases. Presence of M protein in SP is of prognostic significance and aids in disease monitoring. If detected for the first time after treatment or if it reappears after due course of treatment or does progressively increase, it indicates local recurrence or disease progression. In this scenario, a prompt reassessment and close follow up is warranted [23]. In the present study, M protein was used to monitor the disease.

SPs are usually diagnosed by lesional biopsy or fine needle aspiration technique. Flouroscopy-guided or CT-guided lesional biopsy of the spine can be performed. Keeping in mind the rarity of SPs, histopathological evaluation is mandatory for diagnosis as well as to rule out other differentials such as bone tumors and lymphomas [14]. In the present study, there were various clinical and morphological differential diagnosis that varied from inflammatory to lymphomas to primary bone tumors. In addition to the morphological evaluation, IHC aided in differentiating plasmacytomas from inflammatory processes that were rich in plasma cells. When required, monoclonality in these lesions was established using kappa and lambda markers that clinched the diagnosis of SPs. MALTomas that showed plasmacytic differentiation of lymphoid cells were excluded using IHC markers where MALT lymphomas were CD20+, CD79a + and SPs were typically CD20 negative.

Patients suspected with SP should have evaluation of bone marrow, with aspiration and trephine biopsy to rule out MM by evaluation for plasma cell morphology and extent of infiltration by these neoplastic cells. The degree of infiltration should be evaluated either by immunophenotyping using BM aspiration or IHC on biopsy to rule out MM. Any tissue biopsy showing monoclonal plasma cells should undergo a repeat bone marrow evaluation to rule out progression to MM [11]. In the present study, all cases that had bone marrow involvement at the time of diagnosis were excluded.

SPs are treated with either radiotherapy, surgery or a combination of both. The option of treatment is site specific with most SPB and SEP of lower respiratory tract being managed with radiotherapy alone. SEPs of upper respiratory system may require both radiotherapy and surgery, whereas, SEPs of gastrointestinal tract require surgical excision [7, 13]. Similar modalities were used in the treatment of the patients in our study. Most of the reported cases in the present series have responded well to surgical excision irrespective of adjuvant chemotherapy or radiation therapy. Recurrences and disease progression to MM are common in SPs and hence every case has to be followed up with radiological and M protein evaluation after completion of treatment [23]. Due to lack of follow up of many cases, the progression of SPs to MM was not performed in our setting.

## Conclusion

The true incidence of Solitary Plasmacytoma in India still remains unraveled, as only a very limited number of studies have been documented. We report 29 cases of SPs with a wide age range and male preponderance. When compared SPBs presented at a later age to SEPs and histopathological examination remains the gold standard in diagnosing these lesions. The diagnosis of MSP requires meticulous investigation to differentiate it from MM. The option of treatment for SPs is site specific with either radiotherapy, surgery or a combination of both. We conclude that the diagnosis of solitary bone and soft tissue plasmacytomas is fairly straightforward and a biopsy is indicated at the earliest in suspected cases. A systematic approach is required for the optimal diagnosis and management of these tumors. Moreover, the increased risk of transformation of SPs into disseminated diseases necessitates periodic surveillance.

## Data Availability

The datasets used and/or analysed during the current study are available from the corresponding author on reasonable request.

## Abbreviations

EMA: Epithelial Membrane Antigen
FISH: Fluorescent In-situ Hybridization technique
IHC: Immunohistochemistry
MGUS: Monoclonal Gammapathy of Undetermined Significance
MM: Multiple Myeloma
PCR: Polymerase Chain Reaction
SEP: Solitary Extramedullary Plasmacytoma
SPB: Solitary Plasmacytoma of Bone
